# Mendelian neurodegenerative disease genes involved in autophagy

**DOI:** 10.1038/s41421-020-0158-y

**Published:** 2020-05-05

**Authors:** Eleanna Stamatakou, Lidia Wróbel, Sandra Malmgren Hill, Claudia Puri, Sung Min Son, Motoki Fujimaki, Ye Zhu, Farah Siddiqi, Marian Fernandez-Estevez, Marco M. Manni, So Jung Park, Julien Villeneuve, David Chaim Rubinsztein

**Affiliations:** 1 Department of Medical Genetics, Cambridge Institute for Medical Research, Cambridge, CB2 0XY UK; 2 UK Dementia Research Institute, The Keith Peters Building, Cambridge Biomedical Campus, Hills Road, Cambridge, CB2 0XY UK

**Keywords:** Macroautophagy, Mechanisms of disease

## Abstract

The lysosomal degradation pathway of macroautophagy (herein referred to as autophagy) plays a crucial role in cellular physiology by regulating the removal of unwanted cargoes such as protein aggregates and damaged organelles. Over the last five decades, significant progress has been made in understanding the molecular mechanisms that regulate autophagy and its roles in human physiology and diseases. These advances, together with discoveries in human genetics linking autophagy-related gene mutations to specific diseases, provide a better understanding of the mechanisms by which autophagy-dependent pathways can be potentially targeted for treating human diseases. Here, we review mutations that have been identified in genes involved in autophagy and their associations with neurodegenerative diseases.

## Introduction

The original definition of autophagy (Greek, “self-eating”) is the delivery of cytoplasmic cargoes to the lysosome for degradation. This process is conserved in all eukaryotic organisms, occurs at basal levels in nearly all cell types, and is tightly regulated by diverse intracellular and extracellular cues. Autophagy is mediated by autophagy-related genes required for the efficient formation and maturation of autophagosomes (double-membraned structures) that are targeted for degradation and recycling after fusion with lysosomes.

**Fig. 1:**
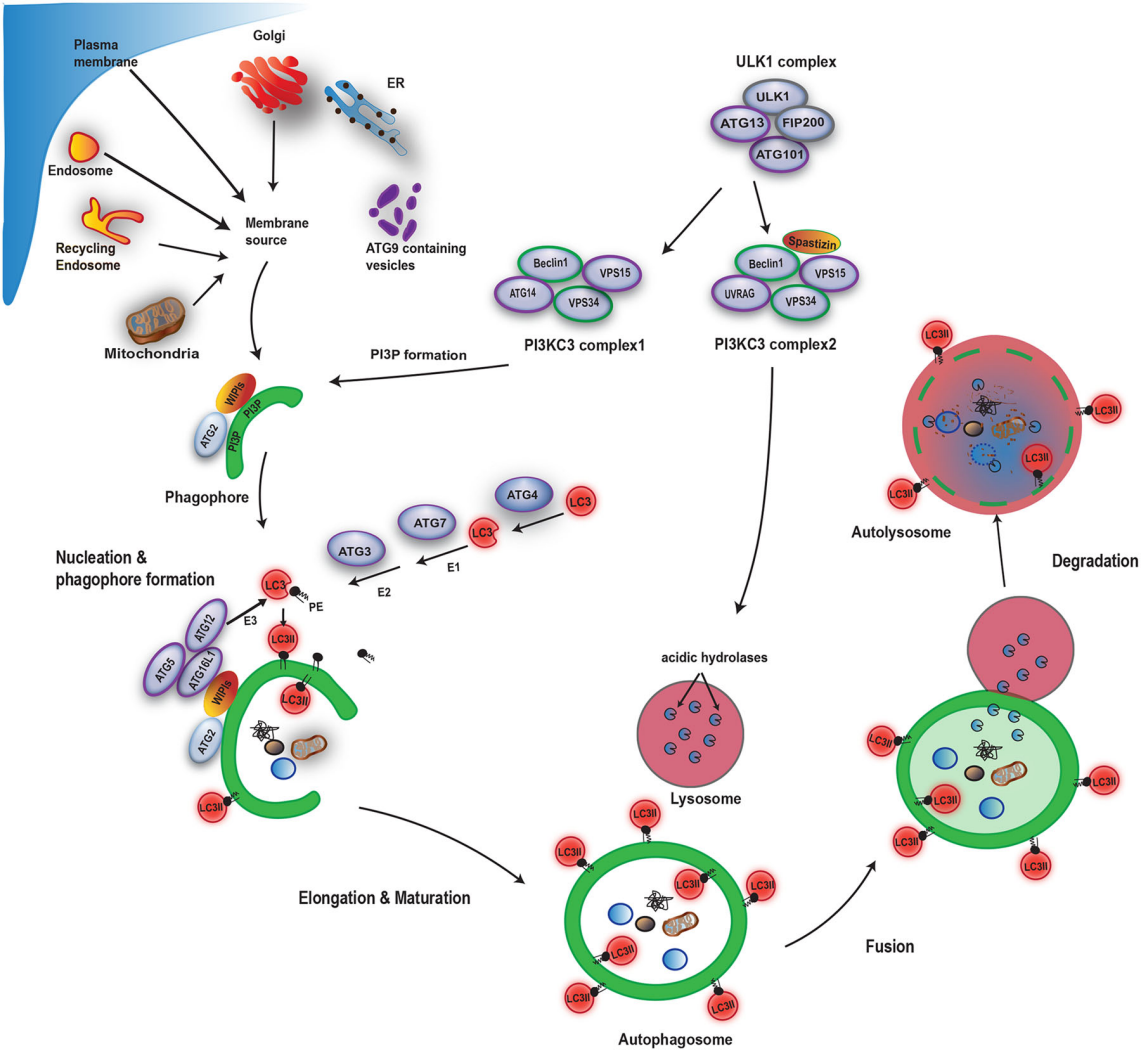
Autophagy overview. The ULK1 complex activates the PI3KC3 complexes, resulting in PI3P synthesis and the nucleation of pre-autophagosome structures that may receive membrane from multiple sources, such as the ER, Golgi, plasma membrane, and recycling endosomes. PI3P is the recruitment signal for WIPI proteins and the ATG12–ATG5–ATG16L1 complex, both are required for autophagosome membrane expansion and completion. Subsequent fusion to lysosomes results in degradation of autophagosomal contents.

The first morphologically recognizable autophagic structures in mammalian cells are cup-shaped double membraned structures called phagophores. After the edges of the phagophores extend and fuse, they become autophagosomes. At the initial steps of autophagy, the ULK1 kinase complex (comprising of ULK1, FIP200, ATG13, and ATG101) plays a major role by phosphorylating multiple downstream effectors^1^. Two distinct Beclin 1/class III phosphatidylinositol 3-kinase (PI3KC3) complexes generate phosphatidylinositol 3-phosphate (PI3P) to act in autophagosome nucleation (Complex 1: Beclin 1, VPS34, VPS15, and ATG14), or endolysosomal and autophagolysosomal maturation (Complex 2: Beclin 1, VPS34, VPS15, and UVRAG). WIPI (WD repeat domain phosphoinositide-interacting) proteins together with ATG2, function in the early stages of membrane elongation at the site of PI3P generation. Autophagosome membrane expansion and completion involves the formation of the ATG5-ATG12-ATG16L1 complex and LC3/GABARAP family protein lipidation. The core ATG proteins are necessary, but not sufficient for degradative autophagy (Fig. 1). Autophagosomal degradation cannot proceed without successful fusion to functional lysosomes. Research in the past decade has unmasked several key factors required for autophagolysosomal fusion, such as the GABARAP subfamily, as well as the CORVET–HOPS and the STX17–SNAP29–VAMP8/VAMP7 SNARE complexes^2–5^.

**Fig. 2:**
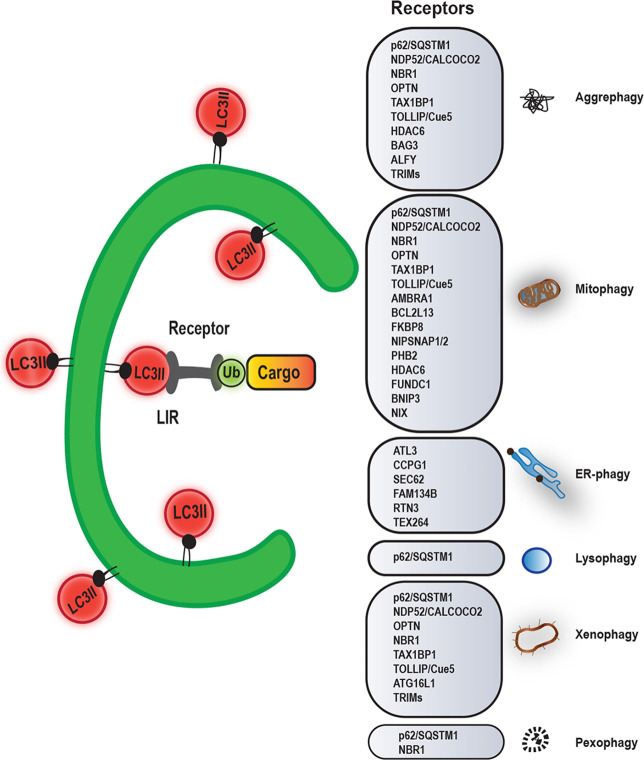
Autophagy receptors and selective autophagy. Through the action of autophagy receptors, specific cargoes are recognized and incorporated into autophagosomes for selective degradation^180^. Autophagy receptors can therefore mediate aggrephagy, mitophagy, pexophagy, ER-phagy, lysophagy, xenophagy, or pexophagy. While this figure aims to provide an overview of the many autophagy receptors involved in different forms of selective autophagy, we have focussed in the text on the most important examples involved in Mendelian neurodegenerative diseases, like P62, ALFY, and OPTN.

Autophagy was initially considered as a bulk degradation process, which lacks substrate specificity. However, we now know that there can be specificity in controlling the choice of substrate that will be degraded by autophagosomes. Numerous studies have reported processes enabling the preferential degradation of aggregation-prone misfolded proteins, such as those involved in the pathogenesis of neurodegenerative diseases, and various organelles, such as damaged mitochondria (Fig. 2)^6–9^. Substrates either contain an LC3-interacting region (LIR) motif that directly binds LC3 or they are labeled with ubiquitin, which is recognized by receptor proteins (also called autophagy adaptors). Receptor proteins contain both an ubiquitin-binding domain and a LIR motif, thus serving as a bridge between the substrates and the LC3 conjugated to phagophores (Fig. 2). Some autophagy adaptors/receptors, such as the autophagy-linked FYVE protein (ALFY) and optineurin, have a dual role as they can bind cargoes, but they also regulate the recruitment of the ULK1 and PI3KC3 complexes to initiate autophagosome formation^10–13^.

Autophagy is required for normal maintenance and function of the mammalian nervous system^14,15^. Multiple studies employing tissue-specific knockout mouse models of core autophagy genes have revealed that autophagy plays a key role in clearing aggregate-prone proteins associated with neurodegeneration and its impairment results in reduces survival and causes progressive neurodegeneration across broad areas of the central and peripheral nervous system^14,15^. Here, we review the known genetic mutations of molecules involved in bulk and selective autophagy and their associations with neurodegenerative diseases (summarized in Table 1).

**Table 1:** Summary of genes involved in autophagy and neurodegeneration.

Gene	Role in autophagy and disease	Ref.
ATG5	Core ATG protein required for LC3 lipidation. Childhood ataxia- or cerebral palsy-associated mutations impair its conjugation to ATG12 or its protein expression, resulting in autophagosome formation impairment.	^16–18,20^
VSP15	Core component of the PI3KC3 complex, required for PI3P biogenesis. The L1224R disease-linked mutation leads to defects in PI3KC3 complex assembly and PI3P production.	^23,26^
WIP4	PI3P effector that mediates autophagosome formation. BPAN-associated mutations show defects in autophagy flux.	^27,28,31,35^
p62/SQSTM1	Autophagy receptor. ALS/FTLD-linked mutations impair its binding to substrates or LC3.	^36–39,41,42,45,46,48^
ALFY	Autophagy receptor that is involved in autophagosome nucleation. MCPH-associated mutations lead to loss of ALFY’s activity.	^10,49,50,52,53^
Parkin	E3 ligase important for mitophagy. Early-onset PD-associated mutations mostly affect its E3 ligase activity	^54,181^
PINK1	Kinase that activates and recruits Parkin to damaged mitochondria. Most of the early onset PD-associated mutations affect its kinase domain, resulting in loss of function.	^59,60,67,181^
OPTN	Autophagy receptor also involved in autophagosome formation and maturation. The E50K glaucoma-associated mutation leads to increased interaction with TBK1, resulting in loss of proper oligomerization and solubility of OPTN. The ALS-associated Q398X and E478G mutations cause a defect in autophagosome–lysosome fusion due to failure in binding with myosin VI.	^12,13,85,87–90,100,182^
TBK1	Kinase that targets autophagy receptors and other molecules involved in autophagosome nucleation, maturation and fusion to lysosomes. Most ALS-linked mutations result in loss of either a functional kinase or binding to its targets.	^62,84,102–105,107,108^
VPS13D	Important for mitophagy. Disease-associated variants display changes in mitochondrial morphology and distribution.	^111–113^
PEX13	Involved in mitophagy and virophagy. ZS-associated mutations lead to reduced activity or levels of PEX13 result in impaired peroxisome function and mitophagy.	^116–118,120,124,125^
VCP	Required for autophagosome maturation. IBMPFD- and ALS-linked mutations impair its chaperone activity or protein expression.	^128–134^
UBQLN2	Binds LC3 and promotes autophagosomal degradation, also regulates lysosomal acidification. ALS-associated mutations impair its ability to bind its partners and result in defects in protein degradation via the proteasome and autophagy.	^135–140^
Spastizin	Regulates ALR, endosome trafficking and autophagosome–lysosomal fusion. SPG15-linked mutations lead to loss of a functional protein, resulting in impaired autophagosome maturation and degradation.	^145,146,148,150–153^
Spatacsin	Regulates ALR and endosome trafficking. SPG11-associated mutations result to loss of function and defects in lysosomal degradation.	^146,148–150^
TECPR2	LC3-binding protein, probably involved in autophagosome formation. SPG49-linked mutations result in protein loss or instability.	^154–156^
EPG5	Tethering protein that regulates autophagosome–lysosomal fusion. Vici syndrome-associated mutations result in loss of function, leading to autophagy impairment.	^160–166^
VSP11	Core CORVET–HOPS complex subunit that regulates autophagosome–lysosomal fusion. gLE-linked mutations result in protein instability and defects in autophagosome degradation.	^168–170^
SNX14	Most likely required for lysosomal function. SCAR20-associated mutations lead to the loss of a functional protein, resulting in lysosomal impairment and defective autophagosome degradation.	^174,175,177–179^

## Autophagy related 5 (ATG5)

ATG5 is a core autophagy protein that functions in autophagosome formation and is required for both of the ubiquitin-like conjugation systems involved in LC3 lipidation. ATG5 is first irreversibly conjugated to ATG12 by the E1-like ATG7 and E2-like ATG10^16^. Then, the ATG5–ATG12 conjugate non-covalently interacts with ATG16L1 to form the ATG5–ATG12–ATG16L1 complex, which functions as an E3-like enzyme for the covalent conjugation of the C-terminal glycine of LC3 to phosphatidylethanolamine (PE), resulting to LC3-II formation^17^ (Fig. 1).

Whole-exome sequencing of two Turkish siblings with childhood ataxia associated with hypoplasia of the cerebellar vermis identified a homozygous missense mutation (E122D) in *ATG5*^18^. E122 is located in the vicinity of the ATG12–ATG5 interaction surface and the E122D mutation impaired the conjugation of ATG5 to ATG12. In patient‐derived lymphoblastoid cell lines, the levels of ATG5–ATG12 conjugate is severely reduced, therefore leading to impaired autophagosome formation^18^. Ataxia is a common clinical sign of impaired coordination of movement and balance during voluntary activity, mainly caused by dysfunction of the complex circuitry connecting the basal ganglia, cerebellum, and cerebral cortex. Dysfunction of the cerebellar vermis causes staggering gait, asynergic movements, dysmetria, nystagmus, intentional tremor, and difficulty in forming speech^19^.

SNPs and variants causing dysregulation of *ATG5* expression levels have also been reported. A homozygous *ATG5* variant (rs6568431 AA genotype) resulting in lower ATG5 expression was identified to be associated with childhood cerebral palsy (CP)^20^. It will be important to validate these findings in subsequent replication studies. CP is a group of permanent motor disorders, often accompanied by disturbances of sensation, perception, cognition, communication and behavior, epilepsy, and secondary musculoskeletal problems. It is caused by nonprogressive disturbances that occurred in the developing fetal or infant brain^21^. A heterozygous *ATG5* variant, located in the promoter region (106774459T > A), which leads to increased expression levels of ATG5, was identified in one Parkinson’s disease (PD) patient^22^. However, it is unclear how and if this variant contributes to the PD pathogenesis.

## Vacuolar protein sorting-associated protein 15 (VPS15)

VPS15 is the regulatory subunit and membrane targeting factor of the PI3KC3 complex, which regulates the production of PI3P^23^. VPS15 and VPS34 together with beclin 1 form three distinctive PI3K complexes. These core components together with ATG14L form the PI3KC3 complex 1, which is essential for the induction of canonical autophagy. In PI3KC3 complex 2, ATG14L is replaced by UVRAG, and the activity is directed towards regulation of endocytosis and endosome fusion, and in complex 3 Rubicon binds to UVRAG to negatively regulate the activity of this complex^24,25^.

A VPS15 missense mutation (L1224R) has been identified in a patient that presented with severe cortical and optic nerve atrophy, localized cortical dysplasia, intellectual impairment, spasticity, ataxia, psychomotor delay, muscle wasting, pseudobulbar palsy, a mild hearing deficit, and late-onset epilepsy^26^. Human fibroblasts carrying the L1224R mutation have an accumulation of autophagy substrates, as well as reduced levels of VPS15, VPS34, and beclin1^26^, indicating that the PI3KC3 complex assembly and therefore PI3P production is impaired.

## WD repeat domain phosphoinositide-interacting protein 4 (WIPI4)

WIPI proteins (WIPI1–WIPI4) are the only known PtdIns3P-binding proteins with conserved PI3P effector functions that recruit downstream regulators and facilitate the formation of autophagosomes^27^. WIPI4 associates with ATG2, a lipid-transfer protein that functions in autophagosome expansion and closure^28^. The recruitment of ATG2–WIPI4 complex onto phagophores requires ATG9 and the TRAPPIII complex that is required for autophagosome closure^29^.

Mutations of WIPI4 (encoded by the *WDR45* gene), resulting in truncated proteins that are prone to degradation, were found to cause an X-linked dominant subtype of neurodegeneration with iron accumulation (NBIA), known as BPAN (beta-propeller protein-associated neurodegeneration)^30–34^. Central nervous system (CNS)-specific *Wdr45* knockout mice recapitulate some phenotypes of BPAN and show ubiquitin-positive protein aggregates and p62/SQSTM1 accumulation in neurons, particularly in axons, suggesting a blockage of autophagy flux^35^. Lymphoblastoid cell lines derived from BPAN patients showed impaired autophagy flux and accumulation of LC3-positive autophagosome membranes that were also positive for ATG9^31^. BPAN patients are characterized by neurological movement disorder, progressive degeneration of the nervous system, and strong iron deposition in the substantia nigra (SN) and globus pallidus (GP).

## Sequestosome 1 (SQSTM1; also known as p62)

p62/SQSTM1 is a multifunctional scaffold protein, which plays important roles as an adaptor/receptor for selective autophagy, but it also mediates activation of the mammalian target of rapamycin complex 1 (mTORC1) on lysosomes and the Keap1–Nrf2 pathway, a major cellular defense mechanism against oxidative stress^36^. Among its multiple domains, Phox1 and Bem1p domain (PB1), LIR, and the C-terminal ubiquitin associated domain (UBA) domains are required for selective autophagy^37–39^, whereas the Kelch-like ECH associated protein 1 (Keap1)-interacting region is required for Keap1 binding^40^.

Several mutations in p62/SQSTM1 have been identified in cases of familial and sporadic amyotrophic lateral sclerosis (ALS) and frontotemporal lobar degeneration (FTLD), leading to loss of function of p62/SQSTM1 in selective autophagy^41,42^, or a decreased interaction with Keap1^43^. In the brains of the ALS–FTLD patients, p62/SQSTM1 is detected in ubiquitin intracellular protein aggregates, suggesting that p62/SQSTM1 and ubiquitin contributes to the formation of inclusions^44^. The familial ALS-associated P392L mutation^45^, located in the UBA domain of p62/SQSTM1, impairs the binding ability of p62/SQSTM1 to polyubiquitin chains, resulting in loss of basal autophagy^46^. Moreover, the L341V mutation in the LIR domain found in sporadic ALS^47^ impairs its binding to LC3, therefore inhibiting p62/SQSTM1-mediated delivery of ubiquitinated substrates to autophagosomes^48^. Also, the ALS–FTLD-associated p62/SQSTM1 P348L and G351A mutations impair Keap1 binding, resulting in reduced Nrf2 signaling and aberrant expression of oxidative response genes^48^. ALS and FTLD are highly related neurodegenerative disorders on the same pathological disease spectrum. ALS clinical signs include motor neuron degeneration, resulting in progressive muscle weakness and paralysis, eventually leading to fatal respiratory failure. The term FTLD covers a range of clinical syndromes associated by atrophy of the frontal temporal lobes in the brain, characterized by behavioral and personality changes^41^

## Autophagy-linked FYVE protein (ALFY)

ALFY (encoded by the *WDFY3* gene) is a member of the Beige and Chendiak-Higashi (BEACH) domain-containing protein family involved in vesicle transport, membrane fission and fusion events, and autophagy^49^. ALFY is required for the recruitment of ubiquitinated proteins to cytosolic p62/SQSTM1 bodies and nuclear promyelocytic leukemia nuclear bodies (PML-NBs)^10^. ALFY, together with p62/SQSTM1 and interactions with LC3 and the ATG5–ATG12 complex, drives the formation of larger aggregates that are targeted for autophagic degradation^10,11^. In addition to its role as an autophagy receptor, ALFY binds PI3P in the autophagic membrane through its FYVE domain and facilitates the binding of ATG5–ATG12 to ATG16^10^, therefore acting as an autophagosome nucleation inducer.

*WDFY3* is a recently identified disease gene where mutations are seen in autosomal dominant primary microcephaly (MCPH)^50^. Primary microcephaly is a group of autosomal disorders characterized by a reduction in the number of neurons, resulting in a smaller brain volume coupled with a head circumference of at least three standard deviations below the age- and sex-specific means^50,51^. Individuals with MCPH usually have intellectual disability and speech delay, with varying degrees of motor delay^50,51^. The heterozygous mutation R2637W in ALFY, located in the PH domain, is directly related to its ability to remove protein aggregates from the cytosol and flies expressing this mutation have smaller and malformed brains with clusters of cells containing ALFY-positive aggregates^52^. Lack of ALFY activity leads to aberrant activation of the Wnt signaling pathway, resulting in reduced neuronal differentiation and microcephaly^52^. Similarly, another mutation located in the PH domain (G2558S) was found to be associated with intellectual disability and microcephaly^53^. By contrast, a missense mutation in the BEACH domain (R2823W), as well as several de novo pathogenic variants of *WDFY3* leading to haploinsufficiency of the gene, result in macrocephaly and cause an autosomal dominant neurodevelopmental disorder with mild-to-moderate neurodevelopmental delay and intellectual disability associated with deficits in motor coordination, as well as autism spectrum disorders^53^. Interestingly, studies on heterozygous Wdfy3^+/lacZ^ mice showed that Wnt signaling is inhibited^53^. The proposed mechanism for this discrepancy describes that mutations on the PH domain of ALFY would lead to aberrant upregulation of Wnt signaling and microcephaly, whereas truncating variants and mutations in the BEACH domain will lead to downregulation of Wnt signaling and macrocephaly^53^.

## Parkin and PINK1

The E3 ligase Parkin and the serine–threonine kinase PINK1 (encoded by the *PARK2* and *PARK6* genes, respectively) play important roles in the maintenance of mitochondrial quality and the degradation of damaged mitochondria through mitophagy^54,55^. PINK1, located in the inner mitochondrial membrane, is constitutively cleaved in the inner mitochondrial membrane by the presenilin-associated rhomboid-like protein and is degraded by the proteasome^56–58^. However, when mitochondrial membrane potential is disrupted, PINK1 is no longer imported into mitochondria and accumulates on the outer mitochondrial membrane^56,57^. PINK1 on the outer membrane phosphorylates Parkin as well as ubiquitin located on mitochondria, thereby releasing Parkin’s autoinhibition and inducing its E3 ligase activity, as well as promoting its recruitment to damaged mitochondria^59–61^. The assembly of Parkin-mediated polyubiquitin chains on damaged mitochondria initiates the recruitment of autophagy receptors, such as p62/SQSTM1 and optineurin (OPTN), as well as activation of the TANK-binding kinase1 (TBK1)^37,62,63^. PINK1, independently of Parkin, also recruits OPTN and NDP52/CALCOCO2 to mitochondria to activate mitophagy, which also induce the recruitment of the components of the autophagosome formation machinery, such as ULK1 and WIPI proteins^64^.

Parkin and PINK1 mutations are a major cause of early-onset PD^65–70^. Considerations have been raised regarding the roles of Parkin and PINK1 in mitophagy, as studies using primary neurons or neurons derived from induced pluripotent stem cells resulted in several controversies^71–73^. However, studies, using patient derived fibroblasts have revealed aberrant mitochondrial function and morphology^74–78^, indicative of mitophagy dysfunction. Patients with *PARK2* mutations show the typical signs characterizing PD, which are rigidity, bradykinesia, postural instability, and tremor, as well as additional signs such as freezing gait and lower foot dystonia. In contrast to typical idiopathic PD, cognitive dysfunction, hallucination, and REM behavioral disorder are not seen. Patients with *PARK6* mutations show clinical symptoms similar to sporadic PD^79^. However, the accumulation of Lewy body aggregates, a pathognomonic feature of sporadic PD, is not a typical feature of Parkin-associated Parkinsonism^80^.

## Optineurin (OPTN)

OPTN is a highly conserved and ubiquitously expressed cytoplasmic protein that contains several domains including a LIR motif and an ubiquitin-binding region^81–83^. OPTN is an autophagy adaptor/receptor protein that participates particularly in the selective autophagy of protein aggregates/pathological inclusions, damaged mitochondria, and intracellular pathogens^12,62,84–87^. OPTN is recruited by PINK1 to damaged mitochondria and subsequently recruits ULK1 in an ubiquitin-binding dependent manner^12^. OPTN also promotes autophagosomal elongation by mediating the recruitment of the ATG12–ATG5–ATG16L1 complex to phagophores^13^. OPTN also regulates autophagosomal maturation through its interaction with the GTPase Rab1a^88^, and autophagosome–lysosome fusion through its interaction with the myosin VI complex^89,90^.

Mutations and polymorphisms in OPTN have been linked to neurodegenerative diseases, including ALS^91–93^ and glaucoma^82,83,94^. A number of studies have shown that glaucoma-associated OPTN mutations induce defective autophagy and increased cell death both in vitro as well as in vivo (in mice expressing the glaucoma-linked E50K mutation)^95–98^. In ALS, OPTN mutations have pathogenic effects as a consequence of deletions of the exon 4 or 5 or nonsense mutations encoding truncated proteins lacking its C-terminal^85,92,99^. More than 20 ALS-linked missense OPTN mutations have been reported. However, the role of most mutations on disease progression is still poorly understood. The ALS-linked OPTN mutations Q398X and E478G lead to failure in myosin VI binding and Myb 1 (Tom1) targeting, causing inhibition of autophagosome–lysosome fusion^90,100^. Another heterozygous missense mutation in OPTN (V295F) induces ALS-like cellular phenotypes, like Golgi fragmentation and increased ER stress^101^.

## TANK-binding kinase 1 (TBK1)

TBK1 is a ubiquitously expressed protein kinase and contains a serine/threonine kinase domain, an ubiquitin-like domain and two coiled-coil domains (CCD1 and CCD2) that mediate homodimerization^102^. TBK1 has a critical role in autophagy by regulating the phosphorylation of the autophagy receptors OPTN, NDP52/CALCOCO2, and p62/SQSTM1^62,102^. Phosphorylation of OPTN by TBK1 increases its affinity to LC3 and its ubiquitin binding^62,84^. The ALS-associated E696K TBK1 mutant, which is deficient in OPTN binding, fails to be recruited to damaged mitochondria^103^, suggesting that mitophagy by OPTN is mediated by TBK1. In addition, TBK1 phosphorylates p62/SQSTM1 on a residue essential for its role in autophagic clearance^104^. Recent studies have suggested that TBK1 is also critical for autophagy initiation by regulating syntaxin 17 (Stx17) phosphorylation and the formation of FIP200-ATG13 pre-autophagosomal structures^105^. TBK1 has also been implicated in the maturation of autophagosomes into autolysosomes^104^, probably through regulation of the cytoplasmic levels of dynein^106^ that regulates autophagosome transport via microtubules to lysosomes.

Mutations in TBK1 have been shown to cause ALS, FTD, and a combination of both (ALS–FTD). From a number of genetics studies with ALS, ALS–FTD, and FTD patients, more than 40 mutations have been found in the TBK1 gene that cause either a frameshift or a premature stop leading to truncated products^102,107^. Several ALS-causing TBK1 mutations induce protein truncation resulting in loss of the CCD2 domain in TBK1, decreasing its kinase activity^107^. Some TBK1 mutations also result in decreased mRNA and protein levels^107^, which may decrease activation of autophagy receptors, resulting in decreased autophagic substrate clearance, and accumulation of protein aggregates in neurons^108^.

## Vacuolar protein sorting 13D (VPS13D)

VPS13D belongs to the VPS13 family, which in humans comprises of 4 proteins (VPS13A-D*)* that appear to be critical for the non-vesicular transport of lipids between adjacent organelles^109,110^. Studies in *Drosophila* intestinal cells reveal that VPS13D plays an important role in mitochondrial fission and mitophagy through its putative ubiquitin-binding UBA domain^111^. VPS13D functions downstream of the recruitment of the fission factor Drp1 to control mitochondrial fission, and upstream of ATG8A to control mitophagy^111^. However, little is known about the specific molecular function of VPS13D.

Several heterozygous mutations in VPS13D have been identified in families with neurological disorders^112,113^. Patient-derived fibroblasts and muscle biopsies show severe alterations in mitochondrial morphology and energy production^112,113^, indicating accumulation of damaged mitochondria. The disease associated with rare recessive *VPS13D* variants is a novel, complex, hyperkinetic neurological disorder of variable age of onset that can be associated with developmental delay and progressive spastic ataxia or paraplegia. Individuals can present with motor delays and gait instability, as well as cognitive impairment. Individuals have also presented with dysarthria, and eye movement abnormalities. The disorder progresses to spastic ataxia or generalized dystonia, which can lead to loss of independent ambulation^112,113^.

## Peroxisomal membrane protein 13 (PEX13)

PEX13 is a transmembrane protein that forms part of the core of the docking/translocation module in peroxisomes and regulates protein import during peroxisome biogenesis^114,115^. PEX13 loss-of-function impairs the clearance of damaged mitochondria and the degradation of Sindbis virus, without disturbing general autophagy^116,117^. Although PEX13 seems to be important for mitophagy and virophagy, the mechanism of PEX13 in selective autophagy remains elusive.

Mutations in PEX13, lead to Zellweger syndrome (ZS), associated with impaired neuronal migration, neuronal positioning, and brain development^118^. Several mutations has been described in PEX13, including missense, nonsense, deletions, and splice site mutations, that lead to complete or partial impairment in peroxisome function^118–120^. As peroxisomes are involved in the biosynthesis of phospholipids, oxidation of fatty acids and detoxification, patients with ZS accumulate long-chain fatty acids, pristanic acid, phyntanic acid, plasmalogens, and C27-bile acid intermediates^121,122^. Mice lacking PEX13 exhibit many of the clinical features of ZS patients, as they lack morphologically intact peroxisomes and have abnormal mitochondrial distribution and morphologies^117,123^. Fibroblasts from PEX13-deficient mice or patient fibroblasts with PEX13 mutations show increased levels of mitochondrial superoxide and membrane depolarization, suggesting that PEX13 loss-of-function can cause aberrant mitochondrial dynamics and impaired mitophagy^116,117,124,125^. Clinically, ZS patients present with severe hypotonia, ocular abnormalities, such as cataracts or glaucoma, and often show seizures, renal cysts, and hepatic dysfunction^122,126^.

## Valosin-containing protein (VCP; also known as p97 or Cdc48)

VCP is a member of the AAA + -ATPase family of chaperone-like proteins and is ubiquitously expressed in all tissues and throughout the brain^127^. Through its interaction with ubiquitinated proteins, VCP regulates diverse cellular functions, including protein degradation through the proteasome and autophagy^128^. VCP is essential for autophagosome maturation through a yet undefined mechanism. Cells that lack VCP or that express disease-associated VCP mutants have an accumulation of immature autophagic vesicles and fail to degrade autophagic substrates, whereas patient-derived myoblasts accumulate aberrant autophagosomes and endo-lysosomes^129–131^.

Mutations in VCP cause a rare and complex multisystem degenerative disease that manifests with inclusion body myopathy, Paget’s disease of bone, and frontotemporal dementia, as well as ALS^132,133^. VCP disease-associated mutations are found throughout the protein sequence, but mostly reside within the N-terminal and D1/2 domains, which are regions important for substrate and cofactor association, as well as VCP ATPase activity, resulting to either imbalanced cofactor binding or aberrant ATPase activity, as well as reduced protein expression^134^.

## Ubiquilin 2 (UBQLN2)

UBQLN2 is a chaperone protein that delivers ubiquitinated cargo for proteosomal degradation via its ubiquitin-associated (UBA) and UBL domains^135^. However, a role for UBQLN2 in regulating autophagic flux has now also emerged. Initial studies showed that UBQLN2 binds LC3 and cells depleted from UBQLN2 have impaired autophagosome degradation^136,137^. Recent studies in flies showed that Ubqn/UBQLN is required for Torc1/mTORC1 activity and lysosomal acidification through its interaction with v-ATPase and its loss leads to defective autophagy flux^138^. Consistent with these data, depletion of UBQLN1, UBQLN2, and UBQLN4 (UBQLNs) in human cells also cause reduced mTORC1 activity and impaired autophagic degradation^138^.

Mutations of UBQLN2 gene are found in cases of familial ALS and ALS–FTD^135^ and the majority reside within its proline-rich domain that is important for protein–protein interactions^139^. Expression of the ALS-associated UBQLN2 P497H mutation in flies results in impaired autophagic degradation, due to a failure in lysosomal acidification^138^, whereas rat spinal motor neurons expressing the UBQLN2 P497H mutation have decreased LC3 lipidation^140^. Interestingly, expression of the P497H or P506T ALS-associated UBQLN2 mutations in human neuroblastoma cells leads to a deficiency in protein degradation through the proteasome system^139^. Therefore, further work is needed to elucidate the involvement of UBQLN2 in autophagy and disease.

## Spastizin, spatacsin, and tectonin β-propeller repeat-containing protein 2 (TECPR2)

Spastic gait genes (SPG), identified by genetic studies of hereditary spastic paraplegia (HSP) patients, regulate several pathways, including ER function, intracellular membrane trafficking, mitochondrial regulation, myelination, lipid metabolism, and autophagy^141,142^.

Spastizin and spatacsin (encoded by the *SPG15* and *SPG11* genes, respectively) physically interact and have similar subcellular distributions, including endosomes, lysosomes, and ER^143–147^. Spastizin and spatacsin play critical roles in autophagic lysosome reformation (ALR), a mechanism of lysosomal biogenesis that maintains a pool of lysosomes able to fuse with autophagosomes after prolonged starvation^148,149^. However, spastizin and spatacsin are differentially involved in autophagosome–endosome fusion. Both proteins interact with the AP-5 complex and the small GTP-binding proteins Rab5A and Rab11, which regulate endosome trafficking and maturation^146,150^, whereas spastizin interacts with the beclin1–UVRAG complex and regulates autophagosome maturation^151,152^. Interestingly, although spastizin mutations impair autophagosome–endosome fusion, spatacsin loss of function had no effect, implying that only spastizin acts at the intersection between endocytosis and autophagy^150,153^.

Tectonin β-propeller repeat-containing protein 2 (TECPR2) has a C-terminal LIR motif which binds to LC3/GABARAP proteins and acts as positive regulator of autophagy^154–156^. Its association with several trafficking components, including COPII coat proteins, is required for the maintenance of functional ER exit site and efficient ER export in an LC3 binding-depending manner^156^. However, the exact functions of TECPR2 are still unclear.

HSPs are rare inherited disorders characterized by age-dependent neurodegeneration of motor neurons, manifesting with weakness of the legs (paraplegia) and involuntary spasms and muscle stiffness (spasticity). Spastic paraplegia type 11 (SPG11), type 15 (SPG15), and type 49 (SPG49) are classified as complex HSPs, because they involve all four limbs as well as additional features, including abnormalities of the brain, such as a thin corpus callosum^157^. SPG11 and SPG15 are the two most common types of HSP. The onset of symptoms varies greatly. However, abnormalities usually become noticeable in adolescence. So far, more than 100 *SPG11* and *SPG15* mutations have been described, being either nonsense or insertions or deletions leading to a frameshift, suggesting a loss-of-function mechanism. Indeed, mice lacking spastizin or spatacsin develop symptoms consistent with spastic paraplegia^145,149^. The clinical features in SPG11 and SPG15 are often indistinguishable, with distinctive features of early onset Parkinsonism, cognitive impairment, white matter changes, mild cerebellar ataxia, retinal abnormalities, and lens opacities^157–159^. SPG49 is caused by mutated *TECPR2*, resulting in a premature stop codon and expression of truncated, rapidly degraded TECPR2 protein^155^. SPG49 often begins in childhood, starting with weak muscle tone, which gradually worsens, causing difficulty in walking. In addition, affected individuals have moderate to severe intellectual disability and distinctive physical features, including short stature and microcephaly. In SPG49 patients, autonomic neurons are affected, causing abnormalities in involuntary body functions such as heart rate, digestion, and breathing. Patients often experience gastroesophageal reflux, which could lead to life-threatening bacterial lung infections.

## Ectopic P granules protein 5 (EPG5)

EPG5 is involved in autophagosome maturation and fusion to the lysosome^160–162^. EPG5 interacts with Rab7 on late endosomes, co-localizes with LC3-positive autophagosomes and the assembled SNARE complex STX17–SNAP29–VAMP8/VAMP7^163^, which is essential for fusion of autophagosomes to lysosomes^3^. These observations, together with convincing in vitro data, suggest that GTP-bound Rab7 recruits EPG5 to late endosomes, enabling endosome–autophagosome fusion followed by recruitment and assembly of the STX17–SNAP29–VAMP8/VAMP7 SNARE complex^163^. Consequently, EPG5 functions as a tether to specifically direct fusion of autophagosomes and endosomes and subsequent fusion to the lysosome.

*EPG5* mutations cause Vici syndrome^164–166^, a rare, recessive neurodevelopmental disorder with multisystem involvement, involving underdeveloped corpus callosum, cataracts, cardiomyopathy, combined immunodeficiency, developmental delay, hypopigmentation, and failure to thrive^165^. Most of the mutations identified in patients result in a non-functional EPG5 protein due to truncation, frameshift, premature stop codons or splicing errors. Consistent with a role for EPG5 in autophagosome fusion to the lysosome, studies in patient fibroblasts revealed an accumulation of LC3 and autophagic substrates due to impaired autophagic flux^164,165^. Endocytotic uptake and degradation was normal in the patient cells, indicating that lysosomal function is maintained and that the EPG5 mutation affects an earlier step in the autophagosome maturation process^165^. Furthermore, genetic variants of *EPG5* have been associated with altered age of onset in Alzheimer’s disease^167^, consistent with the importance of autophagy in protection against neurodegeneration.

## Vacuolar protein sorting-associated protein 11 (VPS11)

VPS11 is part of the CORVET–HOPS complex, which is essential for fusion of endosomes or autophagosomes with lysosomes^168^. The C846G missense mutation in VPS11 causes aberrant ubiquitination, accelerated turnover of the protein and impaired protein complex stability^169^. A very rare mutation has been also found (reported in two siblings) that involves a 9 amino acid deletion in VPS11 (ΔL387_G395) leading to reduced protein levels^170^. The HOPS complex instability (due to loss or reduced levels of VPS11) is associated with dysfunctional autophagy–lysosome trafficking^169^.

Mutations in VPS11 cause genetic leukoencephalopathies (gLEs), a group of heterogeneous disorders with abnormalities affecting the CNS. Patients affected with gLEs manifest variable neurologic phenotypes, including motor impairment, hypotonia, pyramidal dysfunction, dystonia, and/or dyskinesia, ataxia, seizures, cortical blindness, optic atrophy, and impaired cognitive development^171^. The neuro-imaging features include severe hypomyelination with clusters of membranous cytoplasmic bodies in dermal unmyelinated nerve axons^170^, similar to what it has been seen in classic lysosomal storage diseases.

## Sorting nexin-14 (SNX14)

SNX14 belongs to the PXA–RGS–PX–PXC subfamily, along with SNX13, SNX19, and SNX25.41 Similar to SNX13, SNX14 contains a putative double transmembrane domain including a short cytoplasmic leader sequence and an RGS domain (regulator of G protein signaling)^172^, implying they share similar functions. Different hypotheses have been formulated about the cellular localization and function of SNX14. Although SNX14 is co-expressed with endosomal sorting markers^173^, it is localized in lysosomes, but not in endosomal or Golgi compartments^174,175^. Moreover, a lipid-binding assay using the PX domain of SNX14 shows binding to PI(3,5)P2^176^, the predominant phosphoinositide associated with lysosomes. SNX14 also localizes at ER–lipid droplet contact sites and it may play a role in fatty acid homeostasis, which when perturbed contributes to neurological disease^175,177^. Altogether, these findings support the notion that SNX14 is required for proper lysosomal function and lipid homeostasis.

Mutations in *SNX14* cause autosomal recessive spinocerebellar ataxia 20 (SCAR20), a neurodevelopmental disorder characterized by severely delayed psychomotor development with poor or absent speech, absent gait and cerebellar atrophy. At least 18 different point mutations or deletions have been described, with most resulting in either truncation or complete loss of the SNX14 protein^175^. Fibroblasts from patients carrying a homozygous splice site SNX14 mutation show accumulation of vacuoles with fine nonspecific granular material and electron-dense laminated inclusions^178^, suggesting that loss of function of SNX14 leads to lipid degeneration and structures similar to multilamellar bodies. In addition, SNX14 mutant fibroblasts show abnormal accumulation of p62/SQSTM1-positive structures, indicating defective autophagosome degradation^178^. Consistent with a role in lysosomal function, mutant fibroblasts have reduced levels of cathepsin D^174,175^ and accumulation of cholesterol in lysosomes^179^.

## Concluding remarks

Accumulating evidence indicates that impaired autophagy results in human disease. Here, we have reviewed the genetic variants found in autophagy related proteins and their association to neurodegenerative diseases. These proteins act steps throughout the autophagy itinerary and their loss-of-function results in diverse neurodegenerative conditions, ranging from developmental disorders to motor neuron diseases and dementia-associated conditions. Some of these studies are restricted to individual small families (not large enough to generate significant genetic linkage), or small genetic association studies that do not have genome-wide significance. Also, most of the disease-associated genetic variants have not been characterized in animal models and the only available functional data are from in vitro studies. Hence, additional work is required to provide robust genetic support, although in some cases the functional data are consistent with disease causality. Further studies towards understanding how autophagy can be modulated in normal and disease conditions and how autophagy perturbation leads to such a diverse conditions are fundamental to understand how to treat human diseases. Drug discovery efforts towards the development of pharmacological agents that will restore normal levels of autophagy in human brains are also required. This is a challenging task as we currently have no assay to measure autophagy flux in human brains.
