# Author Correction: Mendelian neurodegenerative disease genes involved in autophagy

**DOI:** 10.1038/s41421-020-0185-8

**Published:** 2020-06-16

**Authors:** Eleanna Stamatakou, Lidia Wrobel, Sandra Malmgren Hill, Claudia Puri, Sung Min Son, Motoki Fujimaki, Ye Zhu, Farah Siddiqi, Marian Fernandez-Estevez, Marco M. Manni, So Jung Park, Julien Villeneuve, David Chaim Rubinsztein

**Affiliations:** 1 Department of Medical Genetics, Cambridge Institute for Medical Research, Cambridge, CB2 0XY UK; 2 UK Dementia Research Institute, The Keith Peters Building, Cambridge Biomedical Campus, Hills Road, Cambridge, CB2 0XY UK

**Keywords:** Macroautophagy, Mechanisms of disease

Correction to: *Cell Discovery***6**, 24 (2020) 10.1038/s41421-020-0158-y

Published online 5 May 2020

In the original publication of this article^1^, the surname Wrobel was spelled incorrectly as Wróbel.
